# Supplementary material for: A new species of Ampharete Malmgren, 1866 (Annelida: Ampharetidae) from Washington and redescription of A. cirrata Webster & Benedict, 1887 and A. labrops Hartman, 1961
Source: PeerJ. 2025 Nov 28;13:e20457. doi: 10.7717/peerj.20457 (PMC12667694; doi:10.7717/peerj.20457)
Supplement: Supplemental Information 1 [file peerj-13-20457-s001.docx]

| **Supplementary Table 1.** Specimens included in the molecular analysis, with museum voucher number, accession number, locality and reference to published sequences. | | | | | | | |
| --- | --- | --- | --- | --- | --- | --- | --- |
|  | **Species** | **Voucher** | **Accession number** | | **Locality** | **Coordinates / Depth** | **Reference** |
|  |  |  | **BOLD** | **NCBI** |  |  |  |
| **1** | *Ampharete californica* | SIO-BIC A9433 | – | MT166986 | Unknown | – | Stiller et al. (2020) |
| **2** | *A. cirrata* | – | – | OM470635 | Baltic Sea | – | Krüger et al. (2022) |
| **3** | *A. cirrata* | – | – | OM470636 | Baltic Sea | – | Krüger et al. (2022) |
| **4** | *A.* *cirrata* | UF 8068 | BBPS506-19 | – | Reads Bay, Washington | 48.49626, -122.82139;  8.5 m | Not published. Specimens revised in this study. |
| **5** | *A.* *cirrata* | UF 8069 | BBPS507-19 | – | Reads Bay, Washington | 48.49626, -122.82139;  8.5 m | Not published. Specimens revised in this study. |
| **6** | *A.* *cirrata* | UF 8071 | BBPS508-19 | – | Reads Bay, Washington | 48.49626, -122.82139;  8.5 m | Not published. Specimens revised in this study. |
| **7** | *A.* *cirrata* | UF 8081 | BBPS509-19 | – | Reads Bay, Washington | 48.49626, -122.82139;  8.5 m | Not published. Specimens revised in this study. |
| **8** | *A. falcata* | ZMBN 89835 | POLNB011-13 | OR891674 | Norway | 60.2691, 5.1157; 98 m | Parapar et al. (2017) |
| **9** | *A. falcata* | ZMBN 89842 | – | OR891679 | Norway | 58.3466, 8.9283; 250 m | Parapar et al. (2017) |
| **10** | *A. falcata* | ZMBN 106563 | – | MG270098 | Norway | 60.2817, 5.20067;  81–68 m | Eilersten et al. (2017) |
| **11** | *A. falcata* | ZMBN 95443 | – | MG270099 | Norway | 58.3466, 8.9283; 250 m | Eilersten et al. (2017) |
| **12** | *A. finmarchica* | SIO-BIC A1100 | – | JX423738 | Norway, Svalbard, Hornsunddjupet | 76.76, 14.95;  291 m | Stiller et al. (2013) |
| **13** | *A. finmarchica* | NTNU-VM 68245 | PONOR080-13 | OR891684 | Norway, Porsangerfjorden | 70.476, 25.397; 107 m | Parapar et al. (2017) |
| **14** | *A. finmarchica* | ZMBN 94828 | POLYNB836-14 | OR891672 | Norway, Barents Sea | 72.30983, 32.34133; 312 m | Parapar et al. (2017) |
| **15** | *A.* *labrops* | UF 8202 | BBPS810-19 | – | Mouth of Kilisut, Port Townsend, Washington | 47.09354, -122.73316;  4 m | Not published. Specimens revised in this study. |
| **16** | *A. labrops* | – | BCPOL392-08 | HM473291 | Canada, Vancouver Island, Bamfield | 48.831, -125.129;  intertidal | Carr et al. (2011) |
| **17** | *A. labrops* | – | BCPOL396-08 | HM473292 | Canada, Vancouver Island, Bamfield | 48.831, -125.129;  intertidal | Carr et al. (2011) |
| **18** | *A. labrops* | – | BCPOL391-08 | HM473290 | Canada, Vancouver Island, Bamfield | 48.831, -125.129;  intertidal | Carr et al. (2011) |
| **19** | *A. lindstroemi* | ZMBN 89905 | POLNB081-13 | OR891673 | Norway, Skagerrak | 57.9282, 9.2809;  247–290 m | Parapar et al. (2017) |
| **20** | *A. lindstroemi* | ZMBN 89844 | POLNB020-13 | OR891681 | Norway, Bergen area | 60.269, 5.116; 98 m | Parapar et al. (2017) |
| **21** | *A. lindstroemi* | ZMBN 95899 | – | OR891685 | Norway, SW coast | 59.03, 5.449; 58–60 m | Parapar et al. (2017) |
| **22** | *A. paulayi* **n. sp.** | UF 7977 | BBPS043-19 | – | North Hood Canal, Washington | 47.83758, -122.62895;  21 m | Not published. Specimens revised in this study. |
| **23** | *A. paulayi* **n. sp.** | UF 7637 | BBPS1016-19 | – | Eastern end of Nisqually Reach, Washington | 47.1706, -122.78051;  109.25 m | Not published. Specimens revised in this study. |
| **24** | *A. paulayi* **n. sp.** | UF 7692 | BBPS1017-19 | – | Eastern end of Nisqually Reach, Washington | 47.1706, -122.78051;  109.25 m | Not published. Specimens revised in this study. |
| **25** | *A. santillani* | ZMBN 98157 | MIWAP520-15 | OR891683 | Morocco, Atlantic | 33.688, -7.614; 55 m | Parapar et al. (2017) |
| **26** | *A. santillani* | ZMBN 98164 | MIWAP527-15 | OR891670 | Morocco, Atlantic | 32.473, -9.274; 40 m | Parapar et al. (2017) |
| **27** | *A. santillani* | ZMBN 115540 | GBAN17713-19 | MG230531 | Spain, Ría de Ferrol | 43.47, -8.23;  shallow subtidal | Parapar et al. (2017) |
| **28** | *A. santillani* | ZMBN 115542 | GBAN17714-19 | MG230532 | Spain, Ría de Ferrol | 43.47, -8.23;  shallow subtidal | Parapar et al. (2017) |
| **29** | *A. undecima* | ZMBN 104774 | – | OR891678 | Norwegian Sea | 63.0372, 4.689;  760–766 m | Parapar et al. (2017) |
| **30** | *A. undecima* | – | – | OR891676 | Norwegian Sea | 62.27, -0.02; 846 m | Parapar et al. (2017) |
| **31** | *A. undecima* | – | – | OR891675 | Iceland, Denmark Strait | 67.868, -23.696;  1,281 m | Parapar et al. (2017) |
| **32** | *Anobothrus gracilis* | ZMBN 95440 | – | MG270106 | Norway, Skagerrak | 57.9282, 9.2809;  247–290 m | Eilersten et al. (2017) |
| **33** | *Anobothrus gracilis* | SIO-BIC A1106 | – | JX423739 | Norway | 63.48, 10.37;  – | Stiller et al. (2013) |
| **34** | *Sabellides manriquei* | – | CMBIA290-11 | – | USA, California | 33.573, -117.985;  57 m | Not published |
| **35** | *S. octocirrata* | SIO-BIC A1109 | – | JX423770 | Norway, Trondhejmsfjord | 63.52, 10.42;  90 m | Stiller et al. (2013) |
| **36** | *S. octocirrata* | ZMBN 89909 | POLNB085-13 | OR891680 | Norway, Skagerrak | 57.928, 9.280; 193 m | Parapar et al. (2017) |
| **37** | *S. octocirrata* | ZMBN 91772 | POLNB459-13 | OR891671 | Norway, W coast | 62.369, 4.463; 192 m | Parapar et al. (2017) |

**References**

Carr CM, Hardy SM, Brown TM, Macdonald TA, Hebert PDN. 2011. A tri-oceanic perspective: DNA barcoding reveals geographic structure and cryptic diversity in Canadian Polychaetes. *PLoS ONE* 6(7): e22232. <https://doi.org/10.1371/journal.pone.0022232>

Eilertsen MH, Kongsrud JA, Alvestad T, Stiller J, Rouse GW, Rapp HT. 2017. Do ampharetids take sedimented steps between vents and seeps? Phylogeny and habitat-use of Ampharetidae (Annelida, Terebelliformia) in chemosynthesis-based ecosystems. BMC Evolutionary Biology 17: 222. <https://doi.org/10.1186/s12862-017-1065-1>

Krüger L, Dietrich A, Bastrop R, Bick A. 2022. From synonym to valid species: Redescription of *Ampharete acutifrons* (Grube, 1860) and *A. cirrata* Webster & Benedict, 1887, and brief descriptions of *A. baltica* Eliason, 1955 and *A. grubei* Malmgren, 1865. *Zootaxa* 5174(4): 357–380.

Parapar J, Kongsrud JA, Kongshavn K, Alvestad T, Aneiros F, Moreira J. 2017. A new species of *Ampharete* (Annelida: Ampharetidae) from the NW Iberian Peninsula, with a synoptic table comparing NE Atlantic species of the genus. *Zoological Journal of the Linnean Society* 183(3): 526–555. <https://doi.org/10.1093/zoolinnean/zlx077>

Stiller J, Rousset V, Pleijel F, Chevaldonn P, Vrijenhoek RC, Rouse GW. 2013. Phylogeny, biogeography and systematics of hydrothermal vent and methane seep *Amphisamytha* (Ampharetidae, Annelida), with descriptions of three new species. *Systematics and biodiversity* 11(1): 35–65. <https://doi.org/10.1080/14772000.2013.772925>

Stiller J, Tilic E, Rousset V, Pleijel F, Rouse GW. 2020. Spaghetti to a Tree: A robust phylogeny of Terebelliformia (Annelida) based on transcriptomes, molecular and morphological data. *Biology* 9(4): 73. <https://doi.org/10.3390/biology9040073>
