# Supplementary material for: A new species of Ampharete Malmgren, 1866 (Annelida: Ampharetidae) from Washington and redescription of A. cirrata Webster & Benedict, 1887 and A. labrops Hartman, 1961
Source: PeerJ. 2025 Nov 28;13:e20457. doi: 10.7717/peerj.20457 (PMC12667694; doi:10.7717/peerj.20457)
Supplement: Supplemental Information 2 [file peerj-13-20457-s002.docx]

| **Supplementary Table 2**. K2P-corrected mean genetic distances (%) between the analyzed species of *Ampharete*, *Sabellides* and *Anobothrus* *gracilis*. | | | | | | | | | | | | | | |
| --- | --- | --- | --- | --- | --- | --- | --- | --- | --- | --- | --- | --- | --- | --- |
|  |  | **1** | **2** | **3** | **4** | **5** | **6** | **7** | **8** | **9** | **10** | **11** | **12** | **13** |
| **1** | *A. cirrata* (Washington) | * | 16.0 | 17.8 | 20.1 | 18.5 | 22.7 | 20.0 | 20.0 | 4.7 | 20.9 | 19.2 | 19.9 | 20.5 |
| **2** | *A. paulayi* **n. sp**. |  | * | 16.1 | 19.4 | 19.6 | 22.0 | 19.5 | 18.0 | 15.4 | 19.8 | 17.5 | 17.8 | 19.9 |
| **3** | *A. labrops* |  |  | * | 21.0 | 17.4 | 20.5 | 16.9 | 18.5 | 18.0 | 21.0 | 19.7 | 19.3 | 19.2 |
| **4** | *A. santillani* |  |  |  | * | 17.8 | 15.3 | 19.8 | 21.0 | 20.2 | 17.4 | 17.8 | 17.8 | 20.9 |
| **5** | *A. undecima* |  |  |  |  | * | 18.9 | 17.7 | 16.9 | 19.1 | 16.1 | 16.9 | 16.4 | 18.3 |
| **6** | *A. lindstroemi* |  |  |  |  |  | * | 21.8 | 20.6 | 22.1 | 21.5 | 20.4 | 20.4 | 22.8 |
| **7** | *A. finmarchica* |  |  |  |  |  |  | * | 17.6 | 20.0 | 18.5 | 19.9 | 19.5 | 19.8 |
| **8** | *A. falcata* |  |  |  |  |  |  |  | * | 20.1 | 18.3 | 18.4 | 17.9 | 18.0 |
| **9** | *A. cirrata* (Baltic Sea) |  |  |  |  |  |  |  |  | * | 20.7 | 18.5 | 19.1 | 20.6 |
| **10** | *A. californica* |  |  |  |  |  |  |  |  |  | * | 14.4 | 15.4 | 17.2 |
| **11** | *Sabellides manriquei* |  |  |  |  |  |  |  |  |  |  | * | 3.4 | 14.7 |
| **12** | *Sabellides octocirrata* |  |  |  |  |  |  |  |  |  |  |  | * | 15.2 |
| **13** | *Anobothrus gracilis* |  |  |  |  |  |  |  |  |  |  |  |  | * |
